# Genetic Mechanisms Underlying the Evolution of Connectivity in the Human Cortex

**DOI:** 10.3389/fncir.2021.787164

**Published:** 2022-01-07

**Authors:** Ewoud R. E. Schmidt, Franck Polleux

**Affiliations:** ^1^Department of Neuroscience, Medical University of South Carolina, Charleston, SC, United States; ^2^Department of Neuroscience, Columbia University, New York, NY, United States; ^3^Mortimer B. Zuckerman Mind Brain Behavior Institute, Columbia University, New York, NY, United States; ^4^Kavli Institute for Brain Science, Columbia University, New York, NY, United States

**Keywords:** evolution, human brain, neuronal connectivity, synapses, human-specific genes, dendritic morphology, human neuron physiology

## Abstract

One of the most salient features defining modern humans is our remarkable cognitive capacity, which is unrivaled by any other species. Although we still lack a complete understanding of how the human brain gives rise to these unique abilities, the past several decades have witnessed significant progress in uncovering some of the genetic, cellular, and molecular mechanisms shaping the development and function of the human brain. These features include an expansion of brain size and in particular cortical expansion, distinct physiological properties of human neurons, and modified synaptic development. Together they specify the human brain as a large primate brain with a unique underlying neuronal circuit architecture. Here, we review some of the known human-specific features of neuronal connectivity, and we outline how novel insights into the human genome led to the identification of human-specific genetic modifiers that played a role in the evolution of human brain development and function. Novel experimental paradigms are starting to provide a framework for understanding how the emergence of these human-specific genomic innovations shaped the structure and function of neuronal circuits in the human brain.

## Introduction

Modern humans possess remarkable cognitive abilities, including abstract reasoning, creativity, and social intelligence. These abilities enabled humans to develop sophisticated technologies, express themselves through language and art, and examine the world through science and philosophy, ultimately culminating into a rapid and ongoing cultural evolution that, for better or worse, sets humans apart from any other species on the planet.

At the timescale of the evolutionary history of life, the saga of human evolution is a relatively short one: modern humans and chimpanzees, our closest living relatives, shared a common ancestor ~6–8 million years ago. This common ancestor then diverged into multiple radiations and extinctions to give rise to *Homo sapiens* and great apes (Prado-Martinez et al., 2013; Moorjani et al., 2016; Besenbacher et al., 2019). In this relatively short time span, the human brain evolved to give rise to one of the most complex biological systems existing today. And while it should be noted that the human brain did not evolve in isolation—other traits, such as habitual bipedalism, manual dexterity, and complex social networks were important contributors to the evolution of the human species (Almécija et al., 2021)—it is ultimately the evolution of the human brain that lies at the heart of our cognitive abilities. How did the human brain evolve to give rise to our remarkable cognition? While this question remains largely unanswered, it is now the object of intense experimental approaches in Neuroscience. Comparative studies performed over the past several decades have provided insights into some of the biological features that sets the human brain apart from those of other species. Moreover, recent progress in genomics and neuroscience has provided an important framework for understanding how human genetic modifiers emerged and how they altered brain development and function.

A seemingly obvious characteristic that makes the human brain stand out is its size, at least when compared to many of the animal models used in research. But when compared to animals such as *Cetacea* or *Proboscidea*, the size of the human brain is exceeded by several species in these orders (e.g., sperm whale brains are six times bigger that those of humans; Manger et al., 2013; Ridgway and Hanson, 2014). Conversely, some bird species, such as those in the *Corvidea* family, rival the intellect of some mammalian species with much larger brains (Nieder, 2017).

Together, these examples illustrate that brain size, while representing an important evolutionary step, is not sufficient to explain the intellectual abilities of a species. Instead, we argue that what is equally critical, but much less well-studied, is how neural circuits are structurally and functionally organized. Indeed, the human brain is not only big, but incorporates specific traits at different levels of circuit organization that sets it apart from other mammals, and in many cases, from its close relatives the non-human primates. In fact, and as we will discuss, the expansion of the human brain, and tangential expansion of the neocortex in particular, presents challenges for the organization of neuronal connectivity, leading to the notion that brain size and neuronal connectivity are inextricably linked.

## Evolutionary Traits of Neuronal Connectivity

Neuronal connectivity is a broad term that encompasses different levels of organization: from chemical synapses, which are the fundamental units of neuronal communication, to local circuit motifs formed by connections between different neuronal subtypes, up to the large-scale neuronal circuit dynamics across the entire brain. Furthermore, each of these levels of connectivity has the potential to shape the structural and functional properties of neural circuits. As we discuss in the next sections, human-specific traits of connectivity have been described for each of these levels of neuronal circuit organization.

### Human-Specific Traits of Synaptic Development

In its most fundamental interpretation, neuronal connectivity refers to the connection formed by two or more neurons. In the mammalian brain, the most prominent subcellular site of this connection between neurons is the neurotransmitter-releasing chemical synapse. This specialized structure is both highly complex—the synaptic proteome has been estimated to rely on the interaction of a core set of ~460 proteins, but may involve between 3,000 to 5,000 different kind of proteins (Sorokina et al., 2020)—and highly abundant: a single mouse layer 2/3 cortical pyramidal neuron (PN) contains on average 7,000 excitatory (E) and over 1,000 inhibitory (I) synapses and in humans has been estimated to be three to four times higher (Eyal et al., 2018; Iascone et al., 2020). And while this number most likely varies dramatically between neuronal subtypes, overall, the human brain is made of >85 billion neurons connected in short-range and long-range circuits by trillions of synapses. Synapses are also highly specialized subcellular structures with different functional and morphological characteristics, such as excitatory synapses formed on dendritic spines of cortical PNs. The neck of dendritic spines functions as a non-linear chemical and electrical filter endowing these E synapses with unique and modifiable properties (Araya, 2014). Considering this high degree of complexity and their critical function in regulating information transfer and various circuit computations, it is perhaps not surprising that synapses have been a substrate for human brain evolution.

Early studies investigating synaptic development in the human cortex found that synaptic development follows a dynamic trajectory with differences between cortical regions and a temporal profile that is characterized by an initial overproduction of synapses followed by synaptic pruning (Molliver et al., 1973; Huttenlocher, 1979; de Courten and Garey, 1982; Huttenlocher et al., 1982; Huttenlocher and Dabholkar, 1997; Jacobs et al., 1997, 2001). This developmental trajectory, with peak synaptic density during early postnatal development, is not unique to humans. However, when compared to non-human primates, the timing of synaptic development is dramatically prolonged in humans. In macaque and chimpanzee, synaptic density of cortical PNs reaches peak levels at around 3 months of age for macaque and three to five years of age for chimpanzee, and reaches adult levels by the end of adolescence (Rakic et al., 1986; Zecevic et al., 1989; Bianchi et al., 2013b). In contrast, in humans the peak is wider with synaptic pruning continuing into the third decade of life (Petanjek et al., 2011). In accordance with these findings, the temporal profile of gene expression is delayed in humans, especially for genes involved in synaptic development (Liu et al., 2012). Human cortical neurons also display increased density of synapses, with up to 40% higher number of synapses per dendritic segment when compared to chimpanzee, macaque or mouse, irrespective of cortical region (Elston et al., 2001; Benavides-Piccione et al., 2002). A comprehensive recent study estimated total number of synapses per neuron in two cortical areas (IT and V1) of 25 species of primates, including humans, and demonstrated that human PNs, compared to any other non-human primates, display a significant increase in synaptic connectivity (Sherwood et al., 2020).

**Figure 1:**
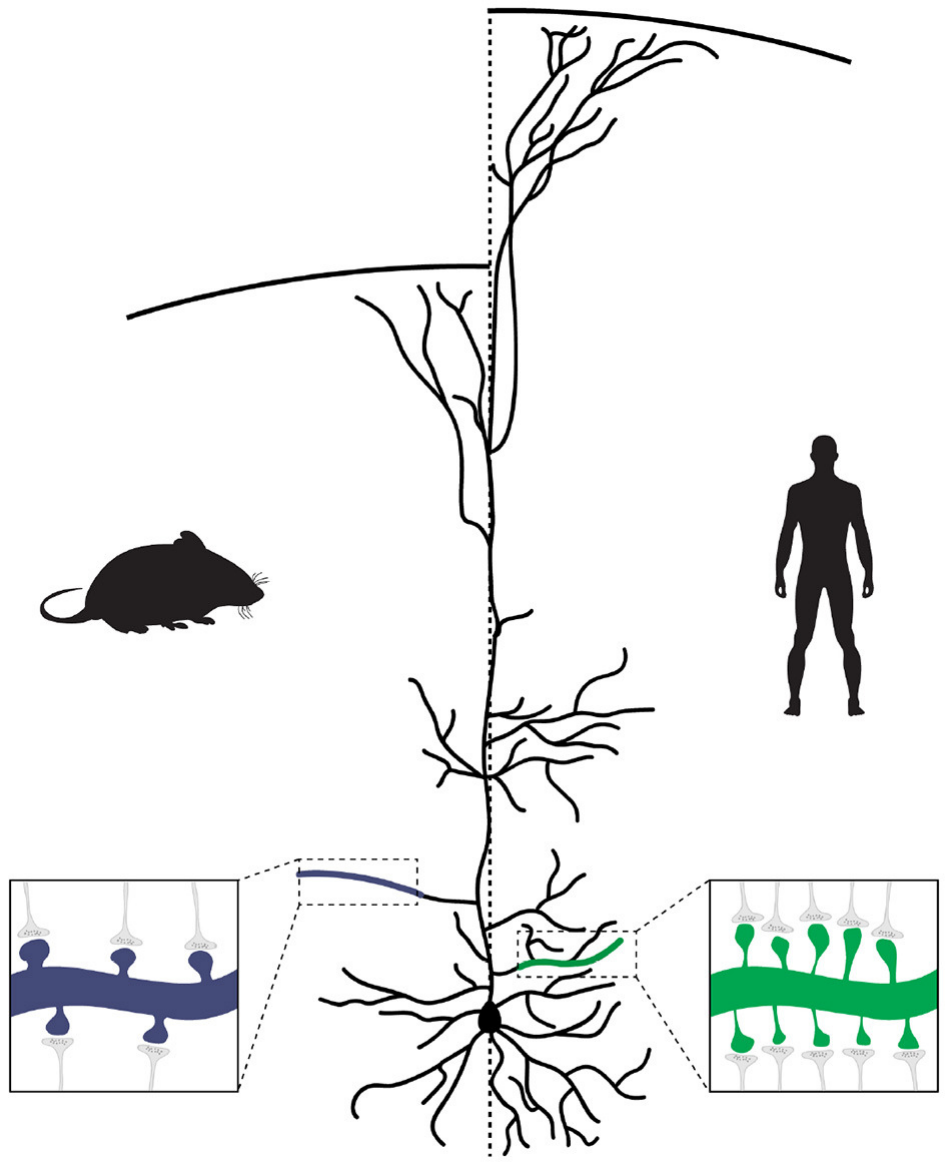
Evolution of pyramidal neuron morphology. In human pyramidal neurons (PNs), a larger number of branch points leads to a more extensive and complex dendritic tree. Each branch also contains a larger number of synapses, resulting in an overall increase in the number of synapses made onto human PNs. In addition, because of the increased cortical thickness of the human brain, the apical dendrites of human PNs extend over a longer distance to reach layer I.

Together with the larger size and complexity of human cortical PNs (as discussed in the next section), this means that connectivity between human neurons, at least in the neocortex, is denser, with each cortical PN receiving many more synaptic inputs from other neurons when compared to other species (Figure 1). In addition, when compared to mouse, human cortical PNs display larger dendritic spines with longer spine necks (Benavides-Piccione et al., 2002), which most likely alters the functional properties of excitatory synapses and the integration of inputs onto human cortical neurons (see Hayashi and Majewska, 2005; Yuste, 2013; Araya, 2014, for extensive reviews on how spine morphology impacts synaptic function).

How delayed synaptic development, increased synaptic density, and altered synaptic morphology impact the function of cortical circuits remains to be determined. As we will discuss, our recent work using mice humanized for the expression of a human-specific modifier suggests that it may dramatically alter cortical circuit architecture, modify neuronal response properties and improve learning (Schmidt et al., 2021). Still, many questions remain. Most studies examining synaptic development in the human brain focused on asymmetric excitatory synapses, with much less known about the developmental profile of inhibitory synapses. In non-human primates, the developmental increase of synapse number and subsequent synaptic pruning predominantly affects excitatory synapses (Zecevic et al., 1989; Bianchi et al., 2013b) and therefore appears uncoupled from the development trajectory of inhibitory synapses. This question is also relevant for synaptic density, although the conserved ratio between excitatory and inhibitory inputs across mammalian species, including humans (Defelipe, 2011), suggests that increased excitatory synaptic density in human cortical PNs is matched by inhibitory synapses in the adult brain. Finally, it remains to be determined whether the underlying functional properties of synapses display human-specific traits. When compared to mice, the postsynaptic proteome of human cortical neurons has a unique composition (Bayés et al., 2012) which together with the changes in morphology of dendritic spines, including longer spine necks (Benavides-Piccione et al., 2002), most likely leads to differences in synaptic function of human neurons. As we will discuss, human cortical neurons display unique physiological properties, but how this relates to the unique molecular signature of human synapses is yet to be determined.

### Dendritic Morphology of Human Neurons

A hallmark of mammalian brain evolution is the expansion of the neocortex. While in the mouse cortical thickness spans, on average, around 1,200 μm, in humans it exceeds 2,500 μm (DeFelipe et al., 2002). Still, the overall layered architecture of the neocortex is largely preserved between mammalian species. This means that, irrespective of brain size, the majority of cortical PNs in both superficial and deep cortical layers, except for those in layer 6, extend their apical dendrite toward the pial surface into layer I, where they connect with long-range top-down inputs from other cortical and subcortical regions (Spruston, 2008; Thomson, 2010). Consequently, human cortical neurons need to extend their apical dendrites over larger distances to reach this layer (Deitcher et al., 2017). This conserved organization of the mammalian neocortex and its increase in size in humans raises the question whether human cortical pyramidal neuron (PN) morphology is conserved and merely scaled up in size, or whether other morphological adaptations emerged in human neurons.

An early study comparing the basal dendrites in the prefrontal cortex (PFC) of human, macaque and marmoset found that human cortical neurons are not only bigger, but that their dendrites are more complex by forming an increased number of branch points (Elston et al., 2001). Similar observations were made in a study that compared human and chimpanzee cortical PNs, showing that besides total dendritic length, overall branching and mean segment length is increased in human cortical neurons (Bianchi et al., 2013a). Interestingly, when compared to elephant, the total dendritic length was similar, but human cortical neurons were more branched with a higher number of dendritic segments (Jacobs et al., 2011). Notably, increased complexity of human PNs is not limited to those located in the cortex, but was similarly observed for CA1 PNs in the hippocampus when compared to mouse (Benavides-Piccione et al., 2020). These studies suggest that a defining feature of human PNs is their more complex and larger dendritic tree (Figure 1). However, these studies were limited to partial reconstruction. To fully investigate the morphology of human neurons, Mohan and colleagues reconstructed and analyzed the complete dendritic tree of 91 human cortical PNs from resected temporal cortical tissue (Mohan et al., 2015). When they compared dendritic morphology to similarly reconstructed mouse cortical PNs, they found that dendritic length in human cortical PNs is significantly increased for all dendritic compartments, especially for the distal segments. The number of branch points was found to be only increased for basal and apical oblique domains. They then used these measures of dendritic morphology from human, macaque (from Duan et al., 2002), and mouse neurons to perform cluster analysis and showed that mouse and macaque neurons clustered together, while most human neurons formed a unique independent cluster.

Together, these findings show that human PNs are not simply scaled up mammalian PNs, but they also evolved more complex dendritic trees. Increased dendritic complexity means an increased number of separate branches that can potentially function as independent compartments. The compartmentalized nature of PNs plays a critical role in how synaptic inputs are integrated (Spruston, 2008) and therefore strongly suggests that the computational properties of human PNs is altered. As we will discuss in the next section, human PNs have distinct physiological properties that may arise from these changes in dendritic morphology, some of which may change their computational properties.

### Physiological Properties of Human Neurons

**Figure 2:**
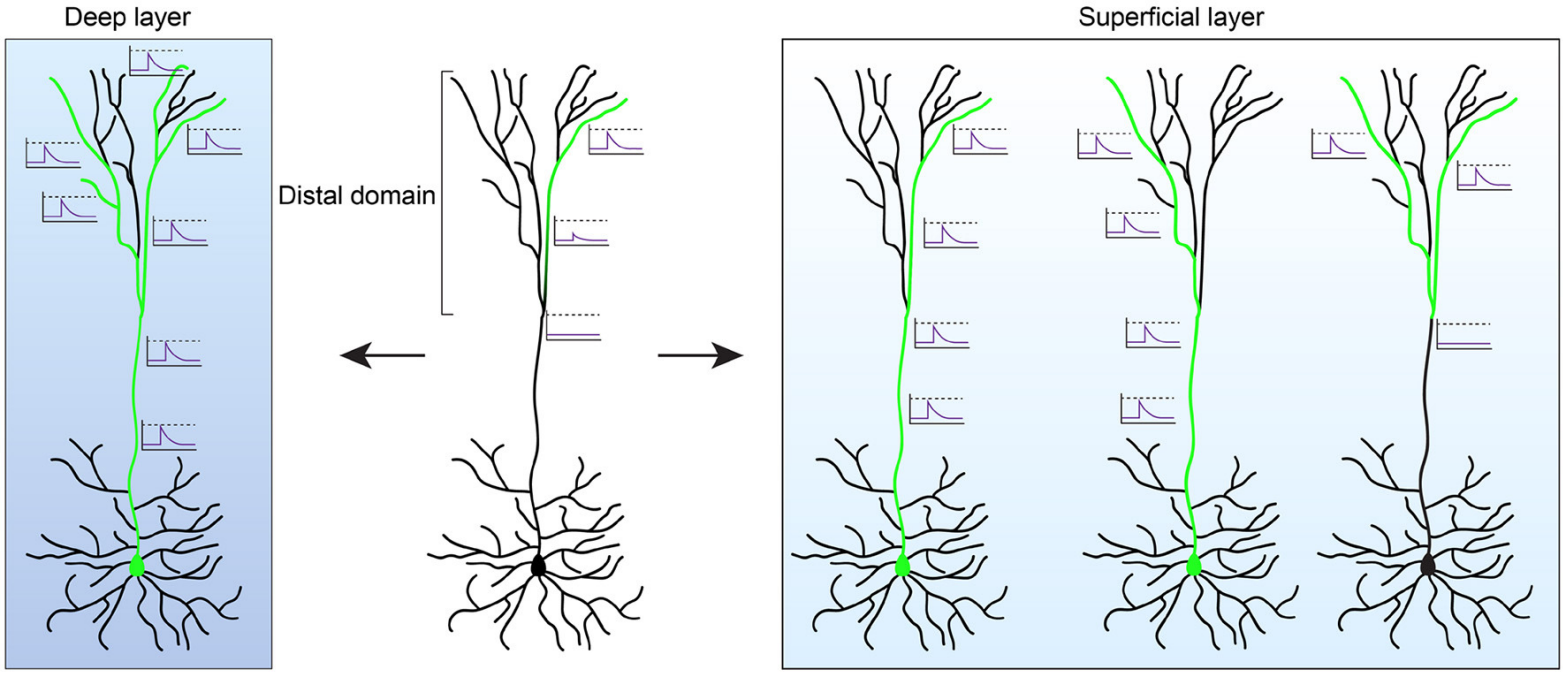
Functional properties of human pyramidal neurons. The length of the apical dendrite of human pyramidal neurons (PNs) can lead to electrical isolation of the distal dendritic domain. In deep layer cortical neurons this results in disrupted coupling between the soma and the distal tuft. The summation of activity in multiple dendritic segments, through non-linear integration rules, are likely required to couple distal activity to somatic spiking (left). As such, the distal domain may function as a parallel computational unit that performs independent computations prior to integration at the soma. In contrast to deep layer cortex, neurons in superficial layers evolved several mechanisms (see main text) that enhances the signaling between the distal domain and the soma (right). In addition, tuning of distal dendrites to specific input strengths enables the distal domain to perform XOR computations, with the summation of activity from multiple dendritic segments leading to suppression of activity.

A key feature of dendritic integration is the transmission of activity—i.e., depolarizing events ranging from synaptic potentials to large dendritic spikes—from the dendrite to the soma where action potentials are generated. However, dendritic depolarization attenuates over large distances, which poses a significant challenge for the increased size of human cortical neurons. The larger distance between soma and distal dendritic segments, especially those of dendritic tufts, would make these compartments electrically isolated, limiting its impact on somatic firing. Indeed, in layer 2/3 human cortical PNs subthreshold potentials from dendrite to soma, and back-propagating action potentials from soma to dendrite, attenuate over much shorter distances than the average length of the apical dendrites (Gidon et al., 2020). Interestingly, human layer 2/3 cortical PNs appear to have evolved specific adaptions to compensate for this (Figure 2). First, human layer 2/3 cortical PNs have a lower specific membrane capacitance than expected. The reduced membrane capacitance means less depolarizing charge and fewer coactivated synapses are required for somatic firing. Together with an increased propagation speed of action potentials, this enhances signal transfer and excitability and compensates for the increased size of human neurons (Eyal et al., 2016).

Second, dendrites of layer 2/3 human pyramidal cortical neurons cortical PNs are more excitable, generating multiple dendritic Ca^2+^ spikes upon current injection. Dendritic action potentials in apical tuft dendrites were also found to be sharply tuned to specific input strengths, with optimization for responses at dendritic rheobase (Gidon et al., 2020). As a result, when inputs exceed optimal input strength, the amplitude of the dendritic Ca^2+^ action potential is reduced. A striking consequence of this change in electrical properties of distal dendrites is that it enables human cortical PNs to execute XOR logical operations in apical tuft dendrites, thereby extending the computational repertoire beyond simple AND/OR operations (Shepherd and Brayton, 1987; Gidon et al., 2020).

Third, HCN1 channels, responsible for the hyperpolarization-activated non-specific cation current I_h_, are expressed in both supra- (layer 2/3 PNs) and infragranular (layer 5/6 PNs) in human cortex, as opposed to mouse where HCN1 expression is mostly restricted to infragranular PNs (Kalmbach et al., 2018). Increased HCN1 expression corresponds with larger “sag” current or rebound responses and less hyperpolarized resting membrane potentials. I_h_ also directly impacts action potentials in response to suprathreshold current injections, leading to increased excitability of layer 2/3 PNs compared to their mouse counterparts, although some of these differences are most likely explained by more than I_h_ alone (Kalmbach et al., 2018). Notably, I_h_ facilitates propagation of synaptic input to the soma and when including I_h_ in a model neuron, the delay between the peak of dendritic and somatic excitatory postsynaptic potentials was decreased, suggesting faster communication between dendrite and soma.

Together, these studies show that human supragranular cortical PNs evolved mechanisms that facilitate communication between distant dendritic compartment and the soma, and that extend the computational operations these neurons can perform on inputs arriving at distal dendrites. Do these findings generalize to other cortical PNs, such as those in deeper layers of the cortex? Surprisingly, human layer 5 cortical PNs display more compartmentalized responses and disrupted coupling between soma and the distal dendritic domain (Beaulieu-Laroche et al., 2018). This in contrast to mouse where in layer 5 PNs of the visual cortex a longer apical dendrite length correlates with increased excitability and facilitates back-propagating action potentials (Galloni et al., 2020). Human neurons at deeper layers of the cortex also exhibit lower excitability when compared to rodents (Beaulieu-Laroche et al., 2018; Kalmbach et al., 2018). These results suggest that human layer 5 PNs are increasingly compartmentalized, with distal dendrites more electrically isolated from the soma. It remains to be determined how this impacts the functional properties of layer 5 cortical neurons. A more isolated apical dendritic compartment, which at its distal regions receives primarily long-range cortico-cortical feedback inputs, may form a separate computational unit that, through non-linear integration of multiple dendritic branches, performs independent computations prior to integration at the soma (Figure 2). As such, superficial and deep layer cortical PNs may have evolved distinct mechanisms in response to the growing size of the cortex that depends on the computational role they play in the circuit. Each of these mechanisms may have enhanced the computational power in distinct ways. By increasing and tuning dendritic excitability of layer 2/3 PNs, distinct logical operations can be performed on a dendritic level that otherwise would require the implementation of a complex neuronal circuit. In contrast, the isolated nature of distal dendrites in deep layer cortical neurons may provide a distinct compartment for parallel processing of information. It remains to be determined how the different layers of the mammalian cortex contribute to the computations performed by cortical circuits, but the distinct evolutionary trajectory of deep and superficial neurons may further hint at their distinct role in the larger circuit.

In addition to changes in physiological properties of dendrites, synapses of human neurons were found to show modified properties when compared to rodent counterparts. The window of spike-timing dependent plasticity in which the coactivation of pre- and postsynaptic neurons leads to strengthening of synapses, appeared wider in human hippocampal and cortical neurons (Testa-Silva et al., 2010; Verhoog et al., 2013). Moreover, in cortical neurons, when the timing of postsynaptic activity preceded that of presynaptic activity, plasticity rules were inverted, leading to weakening of synapses, an effect not observed in the rodent brain (Verhoog et al., 2013). Synapses of human cortical PNs were also found to be mostly depressing with much faster recovery times than those observed in rodents, allowing them to track inputs at higher rates and encode information at a higher bandwidth (Testa-Silva et al., 2014). And synapses of neurons across layers of the temporal lobe contain a larger pool of synaptic vesicles when compared to rat (Yakoubi et al., 2019a,b; Schmuhl-Giesen et al., 2021).

A subset of synapses between cortical PNs and inhibitory neurons are also unusually strong in humans (Molnár et al., 2008). Termed very large excitatory postsynaptic events (VLEs), these strong synapses enable a subset of cortical PNs to drive, with a single spike, the postsynaptic inhibitory neuron to fire. Single spikes were also found to propagate through the circuit by initiating complex poly-synaptic cascades (Molnár et al., 2016; Szegedi et al., 2016). In addition, VLEs are highly plastic, with repeated activation quickly leading to long-term depression (LTD) (Szegedi et al., 2016). VLEs may be important drivers for the formation of neuronal assemblies and, together with their activity-induced plasticity, may provide human cortical circuits with an additional mechanism for rapidly forming and dissolving assemblies important for learning and memory (Holtmaat and Caroni, 2016).

The question remains how the evolutionary changes in the morphology and physiology of human neurons played a role in the emergence of human cognition. Interestingly, some of the described features positively correlate with human cognitive performance, including total dendritic length, dendritic branching and faster action potential rise speed (Goriounova et al., 2018). The ability to integrate information from a larger number of inputs, track these inputs at higher frequencies, and perform a larger repertoire of computations may have represented key evolutionary steps for the emergence of human cognition. However, it should be noted that most studies described here are limited to comparisons between human and rodent. A full description of the physiological features unique to human neurons will require investigation of a larger number of species. In fact, one exciting and recent study compared the biophysical properties of layer 5 PN dendrites in human and 9 other mammalian species and concluded that human layer 5 PNs are complete outliers with regards to the otherwise conserved rules that control the conductance of voltage-gated potassium and HCN channels (Beaulieu-Laroche et al., 2021). Future investigations will need to determine the molecular mechanisms underlying these potentially unique properties of human layer 5 PNs and the consequences on dendritic integration, as well as the circuit coding properties endowed to human cortical circuits.

### Organization of Neuronal Circuits in the Human Brain

As discussed in the previous sections, human cortical neurons are not simply scaled up versions of mammalian neurons. This is similarly true for the human neocortex. When compared to chimpanzee, homologous areas such as primary sensory and motor areas are roughly equivalent in size (i.e., relatively smaller in humans), while cortical association areas are disproportionally larger in humans (Krubitzer and Kahn, 2003; Preuss, 2011; Buckner and Krienen, 2013). Furthermore, parcellation studies of the cortex, based on a variety of measures such as cytoarchitecture, connectivity, and functional activity, indicate that the human brain contains a larger number of distinct cortical regions when compared to mouse, New World and Old World monkeys (Van Essen and Glasser, 2018). In addition, specific cortical pathways known to play a role in language are stronger or unique to humans, including the extension of the arcuate fasciculus into the superior temporal sulcus and middle temporal gyrus, and the strong structural connectivity of the laryngeal motor cortex to somatosensory and inferior parietal cortices (Catani et al., 2005; Dick and Tremblay, 2012; Kumar et al., 2016). Cortical thickness also did not scale up linearly but favored expansion of supragranular layer 2/3, which is mainly involved in cortico-cortical connectivity (Zeng et al., 2012; Krienen et al., 2016).

The increased number of cortical regions, expansion of the superficial cortical layers containing long-range cortico-cortical projection neurons, and additional pathways connecting cortical regions strongly suggests a shift toward increased cortico-cortical connectivity in the human brain. This is supported by the fact that white matter tracts take up over 50% of total brain volume, with white matter volume being disproportionately large in the PFC of humans (Schoenemann et al., 2005). A critical feature of the mammalian neocortex is the strong interconnectivity between brain regions, allowing for hierarchical processing of information and integration of different modalities and internal states. Increased cortico-cortical connectivity facilitating these cortical functions may therefore represent an important underlying mechanism for the emergence of human cognition. However, the increase in overall size of the brain presents a critical challenge: maintaining similar overall connectivity—i.e., maintaining the same number of direct and indirect connections, or “connectivity distance” between brain regions—requires an exponential increase in connectivity. And, due to cortical regions becoming more distant, projections that connect these cortical regions become longer. Spatial constraints and energy demands set limits on the amount of white matter that can be supported, which raises the question whether the increase in brain size, especially that of the neocortex, led to a scaling of wiring that is quantitative (more of the same), or whether the human brain evolved a qualitatively different neuronal circuit architecture.

Results from connectome analyses indicate that brain evolution is characterized by a minimization of physical wiring costs (Bullmore and Sporns, 2012). An extensive survey across 123 mammalian species found that overall connectivity, measured as connectivity distance, is largely conserved between mammalian brains, but that an increase in brain size corresponds to a shift from interhemispheric connectivity to intrahemispheric connectivity (Assaf et al., 2020). Furthermore, work using DTI-based reconstruction of connectivity in humans and chimpanzee, found that in humans connectivity shifted toward increased connectivity between higher-order multimodal association areas (Ardesch et al., 2019). And while connectivity between areas with primary cortical regions was reduced, humans were also found to display increased connectivity between primary and unimodal association areas. Length of long-range projections underlying this connectivity is increased in humans and, using graph theory analysis, were found to contribute significantly more to global network integration when compared to chimpanzee (Ardesch et al., 2019). This may suggest that in the human brain, increasing global network integration through increased cortico-cortical connectivity provided evolutionary benefits that outweighed the cost of increased white matter.

**Figure 3:**
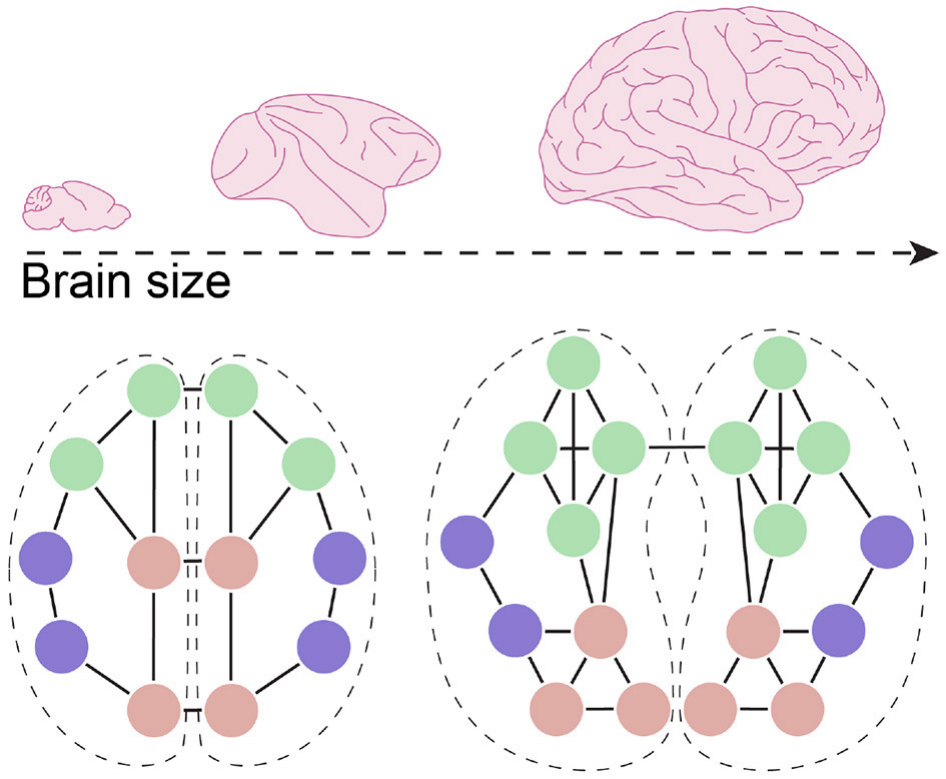
Connectivity changes in larger brains. With an increase in brain size, and the expansion of the neocortex in particular, overall connectivity shifted toward intrahemispheric connectivity with a relative reduction in connectivity between hemispheres. In humans, connectivity between specific domains, such as multimodal areas, also increased, creating more highly connected modules, while long-range projections between modules facilitate global integration of the network.

Together, these data suggest that the expansion of the neocortex corresponded to increased cortico-cortical connectivity while, most likely due to wiring constraints, connectivity shifted to more intra-hemispheric connectivity and a circuit architecture that favors specific long-range connections in order to increase global integration (Figure 3). It should be noted that the concept of local and long-range projections often refers to very different types of connectivity when comparing between species with vastly different brain sizes. For example, neurons that provide local inputs to a layer 2/3 cortical neuron in mouse somatosensory cortex are between 20 and 800 μm away (Schmidt et al., 2021). In contrast, when assessing connectivity in the human brain, voxel sizes are often in the 1 to 2 mm^3^ range, with local connectivity referring to circuits that span millimeters to centimeters—distances that in mice would be considered long-range connectivity. Similarly, the concepts of increased or decreased neuronal connectivity can have different meanings depending on the scale at which we examine these. Brain size is negatively correlated with axon density in the corpus callosum, in agreement with the reduced interhemispheric connectivity described above (Phillips et al., 2015; Assaf et al., 2020). However, due to the increased availability of synapses on postsynaptic human cortical neurons (see previous sections), these axons likely connect with a larger number of neurons. As such, a relative reduction of connectivity between hemispheres, as examined using DTI-based tractography, can still mean that an individual postsynaptic neuron receives inputs from a larger number of individual presynaptic partners and therefore be part of a larger number of cell assemblies. These differences are highly relevant for understanding how changes in connectivity modified information processing in the human brain. While the larger organization of axonal pathways provides information about how information flows across large distances, integration and computation ultimately takes place at the single neuron level. Interestingly, studies analyzing the relationship with macro- and microscale elements of connectivity have shown that some of these measures correlate (Scholtens et al., 2014). Insight into how the connectivity profile of individual human neurons evolved, how it relates to the large-scale organization of neuronal circuits, and how it modified the functional properties of neuronal circuits will be required to fully grasp how neuronal circuits evolved to give rise to human cognition.

### Remaining Questions About the Unique Architecture of the Human Brain

Gaining insight into what aspects of circuit connectivity is unique in the human cortex requires an overall understanding of the fundamental principles that govern neuronal connectivity in the mammalian brain, and how each species, including humans, evolved changes to neuronal connectivity as an adaptation to their ecological niche. As we gain a better understanding of neuronal connectivity in other mammals, we are also better able to direct our inquiries to those aspects of connectivity that may be unique in humans.

For example, it has become increasingly clear that a variety of glial cells, such as astrocytes, oligodendrocytes, and microglia, play an essential role in the formation and function of neuronal circuits (Eroglu and Barres, 2010; Wilton et al., 2019). Interestingly, some of these glial cell types are more diverse and more complex in humans. Protoplasmic astrocytes are over 2.5 times larger in humans and cover over 10 times more synapses when compared to rodents. In addition, distinct morphological subclasses of astrocytes are presents in primates, including interlaminar astrocytes in layer 1 that extend their fibers up to the pial surface and down to layers 2 through 4 (Oberheim et al., 2006, 2009). In humans, these interlaminar astrocytes are more abundant when compared to chimpanzee. And compared to mice, human astrocytes display faster Ca^2+^ wave propagation. As for astrocytes, microglia also appear more diverse in humans. Single-cell transcriptomics across 18 species revealed larger heterogeneity among human microglia, including the potential existence of a human-specific subclass of microglia (Geirsdottir et al., 2019; Prinz et al., 2019). And oligodendrocytes, which play a critical role in myelinization, display accelerated gene expression evolution (Berto et al., 2019). The functional impact of these changes in glial biology, whether these changes evolved in response to changes in neuronal circuits in humans and therefore play a similar role as those in other mammals, or whether they contribute to some of the unique structural and functional traits of neuronal circuits in the human brain, is unclear and will be an important topic for future studies.

Similar questions about the role and evolution of neuromodulatory systems remain unanswered. Overall innervation patterns appear largely conserved, except for increased dopaminergic innervation of the medial caudate nucleus and thalamic subnuclei in humans (García-Cabezas et al., 2007; Raghanti et al., 2008a,b, 2016). However, in contrast to excitatory and inhibitory synapses, those of the different neuromodulatory systems are less well-defined, making it harder to quantify neuromodulatory connectivity in human brain tissue. As underscored by their role in a large number of neurodevelopmental and psychiatric disorders, these systems are critical for proper circuit function and it will be of high interest to uncover the extent to which neuromodulatory systems are conserved and whether they display unique human-specific features in their organization or function.

Most of our knowledge of changes in connectivity in humans relates to cortical PNs. However, a large number of different neuronal cell types exist in mammalian cortex, and even cortical PNs themselves are not a homogenous class of neurons (Hodge et al., 2019). A specific subclass of excitatory neurons has been described for several large-brained mammals, called von Economo neurons, that are larger and more numerous in human, and absent in rodent (Nimchinsky et al., 1995; Allman et al., 2010). These projection neurons have a unique somato-dendritic morphology and are primarily found in layer 5 of the frontoinsular and anterior cingulate cortex. While the presence of these neurons only in these two brain regions may be unique to great apes or even humans, many questions about the connectivity, function, or even clear criteria for their identification is still lacking (Hodge et al., 2020; Banovac et al., 2021). These differences in cell type composition could directly impact the basic circuit motifs of neuronal circuits in the human brain. Studies examining the function of different cell types, and how they integrate into cortical circuits are therefore essential to fully characterize neuronal connectivity in humans.

Finally, most of our current knowledge on human-specific features of brain development and function is derived from comparisons between a limited number of species that are often rodent or monkey, and predominantly from comparative studies performed in select regions of the neocortex. More comprehensive comparative approaches, such as detailed analysis between different cortical layers (e.g., see Gilman et al., 2017), subcortical regions, and a larger variety of species will be required to fully map what features are truly unique to the human brain. Such studies would also provide a more comprehensive understanding of whether and how cortical circuits co-evolved with subcortical brain regions, such as the dorsal thalamus (Halley and Krubitzer, 2019).

## A Search for Genetic Modifiers of Cortical Connectivity in Humans

The evolution of biological features, including human-specific features of neuronal connectivity, relies on the incorporation and stabilization of heritable genetic traits. Over the past decade a wealth of information has been uncovered about the mechanisms that played a role in human brain speciation. A key challenge is to understand how these genetic changes modified molecular pathways and cellular properties to give rise to human-specific features of brain development and function. In the next sections, we will discuss how recent work has provided a framework for understanding the functional context of some of these unique human genetic modifiers and how they shaped neuronal connectivity in the human brain.

### Human-Specific Gene Duplications

Gene duplication represents a major driver of speciation (Ohno, 1999; Hurles, 2004). This includes the *Homo* lineage for which a number of specific segmental duplications have been identified, including those with expression in the developing brain (Bailey et al., 2002; Fortna et al., 2004; Dennis et al., 2017). Although the function for many human-specific gene duplications (HSGDs) remains to be determined, recent work has provided intriguing insight into the role some HSGDs may play in human brain evolution. Two HSGDs, *ARHGAP11B* and *NOTCH2NL*, were recently discovered to promote self-renewal of cortical progenitors in the developing brain, leading to the generation of an increased number of neurons, possibly underlying the expansion of the neocortex in humans (Florio et al., 2015; Fiddes et al., 2018; Suzuki et al., 2018; Xing et al., 2021). Interestingly, NOTCH2NL was found to activate the Notch pathway (Fiddes et al., 2018; Suzuki et al., 2018). This highly conserved pathway plays a critical role in the developing and adult brain, including the regulation of dendritogenesis and structural plasticity (Louvi and Artavanis-Tsakonas, 2006; Ables et al., 2011). The role of *NOTCH2NL* during later stages of development or in adulthood remains unexplored, but if it indeed modifies Notch signaling in postmitotic neurons it may be involved in modifying structural or functional connectivity, perhaps even coupling the expansion of the neocortex to the emergence of specific patterns of connectivity.

**Figure 4:**
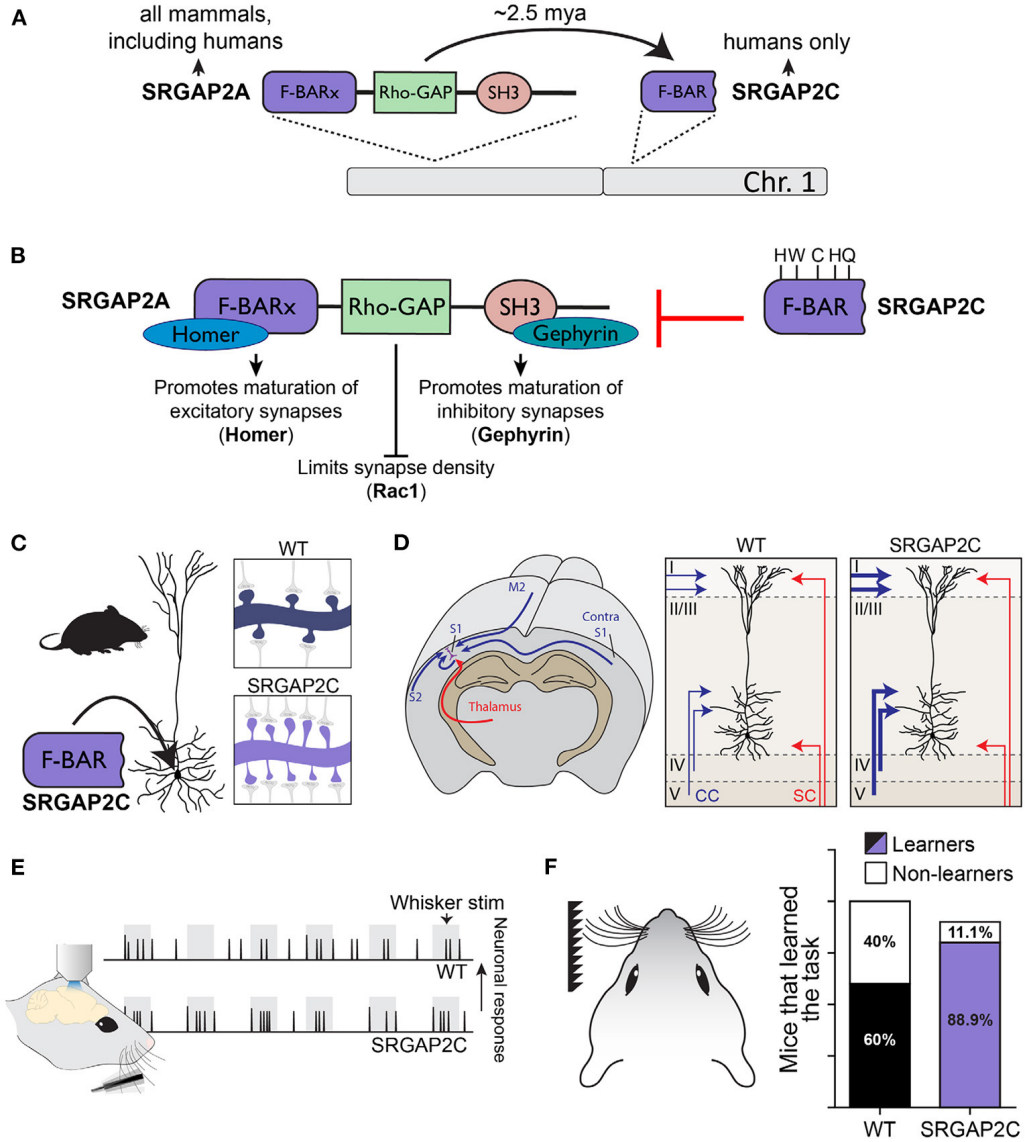
SRGAP2C as a human-specific modifier cortical connectivity and function. **(A)** The ancestral copy SRGAP2A, which is located on chromosome 1, is present in most mammals. Duplication of SRGAP2A in the *Homo* lineage resulted in the emergence of multiple copies, one of which is SRGAP2C. Humans, as the only extant *Homo* species, are now the only species to possess this copy. **(B)** SRGAP2A contains an extended F-BAR domain (F-BARx), through which it binds Homer and promotes the maturation of excitatory synapses. The SH3 domain interacts with Gephyrin to promote inhibitory synapse maturation. The Rho-GAP domain limits synaptic density through Rac1. SRGAP2C is composed of the truncated F-BAR domain of SRGAP2A, with five distinct amino acid changes specific to SRGAP2C. SRGAP2C retains the ability to dimerize with SRGAP2A and inhibits all functions of the ancestral protein. **(C)** Expression of SRGAP2C in mouse cortical pyramidal neurons (PNs) leads to increase synaptic density. **(D)** SRGAP2C-induced changes in synaptic development of cortical PNs leads to a specific increase of local and long-range cortico-cortical inputs. **(E)** Neuronal responses to whisker input of SRGAP2C-expressing mouse cortical PNs are more reliable and more selective to the stimulus. **(F)** Mice humanized for SRGAP2C expression display improved learning in a cortical-dependent whisker-based texture discrimination task. Panel F modified with permission from Schmidt et al. (2021).

A more direct link between neuronal connectivity and HSGDs has been described for Slit-Robo GTPase Activating Protein 2C (*SRGAP2C*). This HSGD emerged as one of several large segmental duplication events affecting the ancestral copy *SRGAP2A* (Figure 4A; Charrier et al., 2012; Dennis et al., 2012). Our work showed that SRGAP2A plays a critical role in regulating the maturation and density of both excitatory and inhibitory synapses (Charrier et al., 2012; Fossati et al., 2016). *SRGAP2C* is a truncated copy of *SRGAP2A* and encodes for a protein containing only the N-terminal F-BAR domain minus the last 49 amino acids. While this makes SRGAP2C intrinsically unstable, it still retains its ability to heterodimerize with SRGAP2A (Charrier et al., 2012; Sporny et al., 2017), which leads to reduced levels of SRGAP2A protein due to proteasome-dependent degradation (Figure 4B; Schmidt et al., 2019). Consequently, expression of SRGAP2C in mouse cortical PNs phenocopies loss of function of the ancestral copy SRGAP2A, resulting in increased density of excitatory and inhibitory synapses and protracted synaptic maturation, features that, as described above, characterize human cortical PNs (Figure 4C; Charrier et al., 2012; Fossati et al., 2016). Moreover, SRGAP2C-incuded changes in synaptic development modify the circuit motif of these neurons by selectively increasing cortico-cortical connectivity, and significantly changes the response properties of layer 2/3 cortical PNs following sensory stimulation by increasing the reliability of their sensory coding features (Figures 4D,E; Schmidt et al., 2021). Strikingly, mice humanized for SRGAP2C expression showed improved learning performance on a sensory discrimination task (Figure 4F). Together these results show how synaptic development provided a substrate for the evolution of neuronal connectivity. By modifying synaptic development, the emergence of SRGAP2C increased cortico-cortical connectivity received by cortical layer 2/3 PNs and changed their functional response properties, which is ultimately associated with improved learning performance. These results also illustrate how the impact of relatively small changes at the subcellular level can cascade through the circuit, leading to large-scale, brain-wide consequences on structural and functional organization of neuronal circuits, an observation that may help explain the relatively short time span over which the human brain evolved.

Even though they were postulated several decades ago as important contributors to human evolution, technical challenges in disambiguating HSGDs from their ancestral copies, and in discriminating between functional copies and pseudogenes, meant that HSGDs have only recently been mapped. Overcoming these challenges represents an important milestone and has opened the door for studying their role in regulating brain development and function, and neuronal connectivity in particular. Other potential modifiers of neuronal connectivity include *CHRFAM7A* which modifies the α7 nicotinic receptor and may converge on the Notch pathway (Gault et al., 1998; Wang et al., 2014; Sinkus et al., 2015; Li et al., 2020), and *SIGLEC11*, a gene duplication with subsequent human-specific gene conversion found in microglia (Hayakawa et al., 2005). Future studies of HSGDs will undoubtedly yield novel and intriguing insights into human evolution and how the unique neuronal circuit architecture of the human brain emerged.

### Human Accelerated Regions as Modifiers of Gene Expression in the Developing Neocortex

Human Accelerated Regions (HARs) are regions of the genome that are highly conserved across species but show a significant excess of human-specific sequence changes, making them prime candidates for encoding biological functions that may have changed during human evolution (Pollard et al., 2006a,b; Prabhakar et al., 2006). Strikingly, most HARs do not code for proteins. Instead, many HARs appear to act as regulatory elements that control how genes are expressed during development. The Noonan lab first discovered that HARs encode human-specific gene regulatory functions, demonstrating that they are likely to direct human-specific changes in the timing, level, and distribution of gene expression, leading to novel cellular and developmental traits (Prabhakar et al., 2008).

HARs are enriched near genes specifically implicated in neocortical development, indicating that they play an especially important role in the expansion and functional elaboration of the human neocortex (Prabhakar et al., 2006; Haygood et al., 2010). Indeed, multiple HARs have been identified that show increased gene regulatory activity in the developing neocortex compared to their homologous sequences in rhesus macaque and mouse (Reilly and Noonan, 2016). Although their role in modifying cortical development remains to be determined, recent studies suggest that HARs regulate genes involved in neurogenesis, axon guidance and synaptic transmission in the developing human cortex, making them potential modifiers of neuronal connectivity in humans (Won et al., 2016, 2019; de la Torre-Ubieta et al., 2018).

### Duplications of Neural Pathways

A hallmark of unique human behavior is the complexity of spoken language. While brain regions involved in human language are not necessarily unique—e.g., homologous regions to Broca and Wernicke were also found in primates (Rilling, 2014)—their underlying connectivity may be more complex in humans. Moreover, while spoken language is unique to humans, complex forms of vocal learning and communication have evolved in other species as well. A common feature between these species is the duplication of motor and auditory pathways, which most likely emerged through convergent evolution (for extensive review, see Jarvis, 2019). Duplication of these pathways, like duplication of genes, allowed these circuits to functionally diverge and be coopted for other behaviors, such as vocal learning.

**Figure 5:**
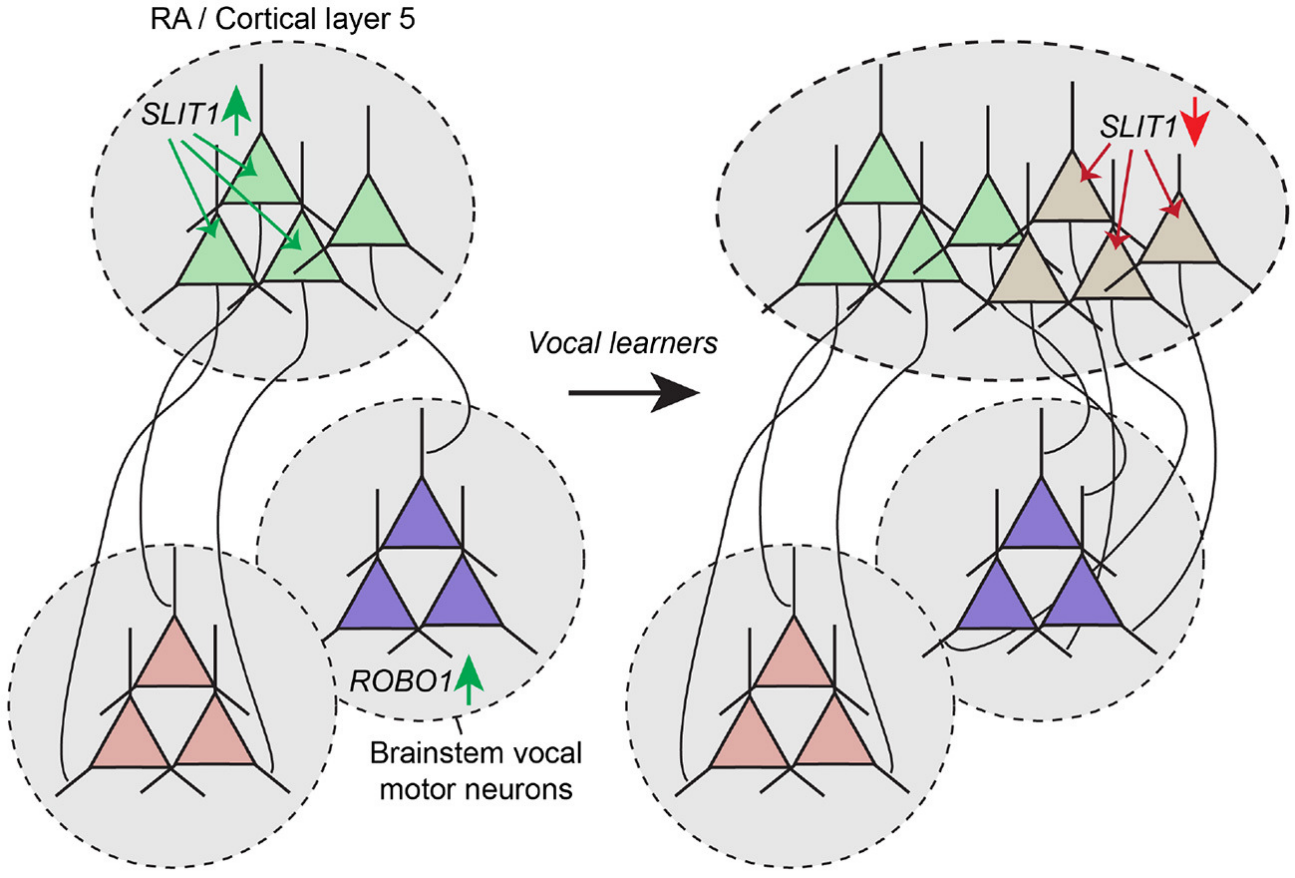
Altered expression of axon guidance molecules mediate rewiring of duplicated motor pathway. Motor neurons in avian RA and mammalian cortical layer 5 express the axon guidance molecules SLIT1. ROBO1 expression in the brain stem region where vocal motor neurons are located prevent SLIT1-positive axons from innervating this area. In vocal learners, duplication of the motor pathway may have resulted in the emergence of RA and layer 5 cortical neurons with lowered SLIT1 expression, enabling these neurons to connect strongly with vocal motor neurons in the brain stem, thereby mediating the emergence of the ability of song production and speech.

An important consequence of this hypothesis is that to become functionally differentiated, the duplicated neural pathway must have some specialization in wiring that functionally sets it apart from the ancestral pathway. Indeed, when analyzing the genetic expression profile of avian song and human language pathways, Pfenning and colleagues found unique expression of multiple genes in these pathways, many of which are known to be involved in the development of neuronal connectivity (Pfenning et al., 2014). One of these genes, *SLIT1*, is an axon guidance ligand that repels axons that express Robo receptors. In songbirds, SLIT1 is downregulated in the nucleus of the arcopallium (RA) of vocal learners, but not in vocal non-learners (Wang et al., 2015). In humans, the ROBO1 receptor is associated with dyslexia (Hannula-Jouppi et al., 2005), and *FOXP2*, a gene critically involved in language in humans, is a transcription factor that directly regulates *SLIT1* (Enard et al., 2002; Chabout et al., 2016). Together these results suggest that evolutionary changes in the SLIT/ROBO axon guidance pathway created a permissive environment for brain nuclei and cortical regions to connect with vocal motor neurons in the brain stem that highly express ROBO1 (Figure 5).

The role of axon guidance molecules in the evolution of neuronal connectivity was previously described for another axon guidance molecule, PlexinA1. In contrast to mice, PlexinA1 levels are low in human layer 5 cortical PNs. When expression of this receptor was lowered in mice, denser connectivity emerged with forelimb motor neurons and mice showed improved performance on a dexterous manipulation task (Gu et al., 2017).

It is important to note that genes involved in axon guidance are not human-specific. However, considering that the evolutionary changes in these genes evolved independently in different vocal learner species, the impact that these changes had on the structure and function of neuronal circuits would have been unique to each species. Implementation of these circuitry changes in an evolving and large primate brain may therefore have had a unique, perhaps human-specific, impact on the complexity of vocal learning.

### Other Mechanisms Underlying Evolution of the Human Brain

In addition to the mechanisms described in the previous sections, a wide range of genetic modifiers likely played a role in human brain evolution. Genetic changes targeting regulatory elements can lead to altered spatiotemporal expression patterns or changes in expression levels of otherwise conserved genes (Kamm et al., 2013; Boyd et al., 2015). For example, hominini-specific (chimpanzee, bonobo, and human) deletion of SOX5-binding sites in Cerebellin2 (CBLN2) enhancers leads to increased levels of CBLN2 expression in the PFC. Moreover, mice carrying the human CBLN2 enhancer 2 show higher levels of CBLN2 expression in the PFC and a PFC-specific increase in synaptic density of layer 2/3 and layer 5 pyramidal neurons (PNs) (Shibata et al., 2021), potentially altering the structure and function of PFC circuits. Enhancers regulating the expression of *FZD8*, a receptor of the Wnt pathway, exhibit human-specific changes that lead to increased cortical and midbrain expression (Boyd et al., 2015). Expression of FZD8 with the human, but not chimpanzee, enhancer resulted in increased cortical size in mice. The Wnt pathway also plays a critical in regulating the assembly of neural circuits and synaptogenesis (Salinas and Zou, 2008; Teo and Salinas, 2021), raising the question whether increased FZD8 expression modifies cortical connectivity. Changes in enhancer sequences were also found in primates for Osteocrin (OSTN) (Ataman et al., 2016). In mice, OSTN is selectively expressed in muscle and bone, but the emergence of three myocyte enhancer factor 2 (MEF2)-responsive elements (MREs) upstream of OSTN results in activity-dependent expression of OSTN in cortical excitatory neurons, which subsequently appears to limit dendritic growth.

## On the Horizon: Technological Developments and Future Directions

Understanding our unique place in the world is a quest that humans have been on for millennia, if not since the dawn of our species. The basis for our cognitive abilities, which enables us to ponder this question in the first place, has especially remained a tantalizing mystery. However, we are now at a unique point in history where we can begin to inquire about the biological underpinnings of cognition and how it evolved in the human lineage over the past ~2.5 million years. This has been facilitated by tremendous technological advances made over the past several decades. Incredible progress in the field of genomics and transcriptomics has allowed for the identification of human-specific genetic modifiers, while access to live human brain tissue has provided unique insight into the physiological, morphological, and gene expression properties of various human neural progenitors and neuronal subtypes. Advances in imaging and data analysis has further provided a unique view into the overall circuit architecture of the human brain. And as previous technological advances have set the stage for our current investigations, we continue to rely on technological developments to drive progress toward a comprehensive understanding of human brain evolution.

A major challenge for studying neuronal connectivity in mammalian brains is the organization at levels that are vastly different in scale. Synapses are sub-micrometer structures while long-range projections in the brain can span centimeters, and even meters for those innervating the periphery. To obtain a complete view of connectivity ultimately requires that we map the entire brain at nanometer resolution, a feature currently out of reach. However, great strides are being made in the development of high-speed multibeam scanning electron microscopes and machine-learning based automated segmentation approaches, which recently enabled the full reconstruction of an ~2 mm^3^ volume of human temporal cortex (Shapson-Coe et al., 2021). And while this is a long way from mapping a full human brain, this data allows for the full analysis of all six layers of the human cortex at unprecedented resolution. The improvements in speed and volume over an earlier reconstruction of 0.13 mm^3^ mouse neocortex (Kasthuri et al., 2015) together with a more recent large scale effort to perform unbiased connectomics analysis in the mouse cortex (Turner et al., 2020), suggests that fully reconstructing an entire mouse brain at nanometer resolution and develop a complete connectomic map of the mouse brain is coming within reach.

Direct access to human brain tissue is invaluable for study the principles of the human brain. However, these approaches are mostly limited to observation without the possibility of experimental manipulations. A particularly interesting approach to study the role of human genetic modifiers in neuronal development and connectivity is the use of xenografts. For this approach, differentiated cortical neurons derived from embryonic stem cells (ESCs) or induced pluripotent stems cells (iPSCs) are transplanted into mouse cortex (Gaspard et al., 2008). This approach allows for the integration of human cortical PNs into the mouse cortical circuit, where they were observed to send out axonal projections and form dendrites with dendritic spines, a feature not observed when cultured *in vitro*. Moreover, morphological and synaptic maturation of transplanted human cortical neurons was significantly prolonged when compared to their mouse neighbors, indicating that protracted maturation is intrinsic to human neurons, in agreement with previous *in vitro* differentiation studies (Matsuda et al., 2020; Rayon et al., 2020), and that they maintain this program even after transplantation (Espuny-Camacho et al., 2013; Linaro et al., 2019). Remarkably, a subset of transplanted neurons were functionally integrated into visual cortical circuits and acquired visual responses such as orientation and direction tuning (Linaro et al., 2019). This work demonstrates that xenograft approaches offer a unique opportunity to study how intrinsic mechanisms unique to human neurons are involved in the formation and function of neuronal connectivity.

The discovery of human-specific phenotypes of neuronal differentiation and connectivity raises the question if and how these genes play a role in the phenotypic expression of disease. We currently have scant knowledge of the contribution of human-specific genes to disease. However, as discussed, the functional characterization of these genes suggests they modify critical pathways involved in neuronal development and connectivity. For example, *SRGAP2C* modifies synaptic development and consequently alters the structural and functional architecture of cortical circuits (Schmidt et al., 2021). In contrast, many genes implicated in autism spectrum disorder (ASD) play a role in synaptic development and function, leading to the notion that ASD should be characterized as a “synaptopathy” (Bagni and Zukin, 2019). Future investigation will need to test whether changes in synaptic development and circuit architecture induced by humanization of SRGAP2C or other human-specific genetic modifiers alter the phenotypic expression of genetic mutations associated with ASD. If this were the case, generating humanized mouse models could drastically change the way we study genes involved in human neurodevelopmental or psychiatric disorders.

In addition, conserved genes involved in synaptic development may have a unique human function. A recent study using induced pluripotent stem cells (iPSCs) to generate human cortical neurons, showed that *NRXN1* deletions lead to impaired synaptic function in human neurons that were not recapitulated in mice (Pak et al., 2015, 2021). Whether this is a direct effect of unique human variation of the NRXN1 gene, or whether this is an indirect consequence of other human-specific features remains unknown. Importantly, it illustrates that human-specific modifiers may uniquely contribute to the cause or phenotypic expression of neurodevelopmental diseases. Establishing approaches that model these human features will be essential to study neurodevelopmental disorders.

Finally, whether through observation or experimental approaches, studying human-specific traits of brain development and function relies on direct comparisons between species to uncover what is general and what is unique. Therefore, in studying unique biological traits of the human brain, we also seek to further understand the fundamental principles underlying species-specific features of brain development in other mammals and non-mammalian species using structural and functional approaches. Only then will we be able to identify features of brain development and function that make us human.

## Author Contributions

ES and FP wrote the manuscript. All authors contributed to the article and approved the submitted version.

## Funding

This work was supported by NIH R01 (RO1NS067557) (FP), an award for the Roger De Spoelberch Foundation (FP), an award from the Nomis Foundation (FP), and NIH K99/R00 (NS109323) (ES).

## Conflict of Interest

The authors declare that the research was conducted in the absence of any commercial or financial relationships that could be construed as a potential conflict of interest.

## Publisher's Note

All claims expressed in this article are solely those of the authors and do not necessarily represent those of their affiliated organizations, or those of the publisher, the editors and the reviewers. Any product that may be evaluated in this article, or claim that may be made by its manufacturer, is not guaranteed or endorsed by the publisher.
